# Family doctor leadership in African primary health care

**DOI:** 10.4102/phcfm.v13i1.3198

**Published:** 2021-09-22

**Authors:** Shabir Moosa

**Affiliations:** 1 Department of Family Medicine and Primary Care, Faculty of Health Sciences, University of the Witwatersrand, Johannesburg, South Africa; 2 WONCA Africa, University of the Witwatersrand, Johannesburg, South Africa

WONCA has initiated the African Forum for Primary Health Care (AfroPHC) [https://afrophc.org/about/] to bring the leadership of the Primary Health Care (PHC) team together to build an accountable team for PHC for well-defined populations at national level in Africa. African Forum for Primary Health Care has grown beyond our wildest dreams in a space of months.

It is unfortunate that there are professional turf issues globally, including Africa. A historical one is that of conflict between doctors and nurses particularly at national levels. The sources of conflict are role boundary issues, scope of practice and accountability.^1^ What has been amazing in developing AfroPHC is the discovery that all cadres of PHC have much in common. It has led to the AfroPHC Statement on Vision and Principles (https://afrophc.org/2020/11/26/afrophc-statement/). Whilst AfroPHC is acutely aware of the shortage of healthcare workers in Africa, the consensus is that all cadres of professions are required in PHC to deliver the African vision of team-based quality care to defined local communities. African Forum for Primary Health Care has showcased what can be achieved at an African level if we practise good leadership and teamwork. See more about AfroPHC (https://afrophc.org/).

Leadership is often cast as an entitled position, usually of authority. A lone African study on physician leadership competencies is useful but teamwork can be seen as superficial friendliness with what are seen as subordinates.^2^ Instead, astute leadership should be robust in exploring various team roles in relation to the task/s. A useful construct is the Belbin Team Role (https://www.belbin.com/about/belbin-team-roles).

The nine Belbin Team Roles are:

- Social Roles
  ■ Resource Investigator: finds ideas to bring back to the team
  ■ Team worker: helps the team gel
  ■ Co-ordinator: focuses on objectives, drawing in team and delegating
- Thinking Roles
  ■ Plant: creative and problem-solving
  ■ Monitor-Evaluator: provides logical eye
  ■ Specialist: brings in-depth knowledge
- Action/Task Roles
  ■ Shaper: brings drive and momentum to task
  ■ Implementer: focuses on workable strategies
  ■ Completer Finisher: scrutinises for errors

The astute leader looks and listens to the team dynamic, seeing how all these roles are being fulfilled in the team, reinforcing the value of each role and trying to fill gaps in the team where these role-players are not available. An astute leader does not need to occupy a formal leadership role to do this. All of the team members should be doing this, in distributed leadership (Chreim et al. 2010, Chreim et al. 2013).^3,5^ However, a person with these skills easily floats to the top and naturally earns the title of team leader within a short while. The nature of future work is not in fixed roles, teams and spaces. The increasingly globalised online world is changing all the time, requiring new spaces, new teams and new roles, even in a primary care practice or clinic.^4,5^

The astute leader does not look for a leadership role but seeks to ensure that the team objectives are being achieved. In doing so, the astute leader assiduously seeks to understand each team member ideas and motivations, both in relation to the initial team objectives and life goals, and then weaves each of these into an overall bigger vision and set of objectives for the team, building a winning coalition of agents.^3^ It is about managing the boundaries between member roles; leadership and clinical roles; formal leaders and other members of the team; different professions; personal life experiences and professional work and the team and what members consider the environment.^5^ Another approach is to manage boundaries and roles around workplace, interpersonal and individual dynamics.^4^ This makes team motivation and cohesion much stronger, reflecting the wise words of Aristotle that ‘the whole is greater than the sum of its parts’.

The really astute leader who goes around places is the one who is not just looking at others’ strengths and current team role but actively seeks to empower others to be leaders like themselves. This kind of leader looks at each person as an asset to make better than they are – building on strengths, helping overcome weakness and seeing in the person the things that they, sometimes, do not see themselves – possibilities. This kind of leader knows that their role in the team is temporary as bigger things beckon. He or she is ready to leave the team and wants to empower a team such that it functions without them.^4^ This kind of leader is more interested in influence rather than control. He or she works with influence knowing that power resides in more than official authoritative roles. It lies in every one of us and beyond. A focus on authority often limits the ability of a real astute leader. This is also the kind of leader that easily climbs the ladder in any setting.

However, content knowledge is also important. It is not necessary to be the most knowledgeable person in the team but an astute leader cannot be clueless about the expertise required. He or she should recognise and defer to the knowledge and expertise required. Organisations should value the perspective of physicians in leadership.^6^ This is, especially, true of family doctors in PHC as PHC is far from simplistic if it is to be delivered well. Leaders in PHC in Africa must acknowledge that clinical governance is required in the PHC team. It is the same respect we have for specialties on a narrow speciality. It is foolish for a family physician to ignore the advice of a dermatologist on how they should be educating a patient on a skin condition and doing health promotion on it. Family physicians are not just knowledgeable in the common conditions across the spectrum of clinical specialties but also in integrating it with a biopsychosocial approach to the patient and a PHC system that engages meaningfully with communities. It is a complex specialty. This means that this hierarchy of clinical knowledge, especially with family medicine training, needs to be acknowledged and respected. Doctors, especially those trained in family medicine to fit into PHC teams in Africa, need to be part of PHC teams and need to be respected for their ability to improve clinical quality in the spectrum of care from a clinic to community, and not just hospital.

We must strive to educate family doctors across Africa in astute leadership such that their leadership is earned quickly. At the same time, we have to advocate for the important role family doctors can play in clinical governance of African PHC in a clinic and community.
